# No Difference between the Sexes in Fine-Scale Spatial Genetic Structure of Roe Deer

**DOI:** 10.1371/journal.pone.0014436

**Published:** 2010-12-28

**Authors:** Nadège Bonnot, Jean-Michel Gaillard, Aurélie Coulon, Maxime Galan, Jean-François Cosson, Daniel Delorme, François Klein, A. J. Mark Hewison

**Affiliations:** 1 Laboratoire Comportement et Ecologie de la Faune Sauvage, Institut National de la Recherche Agronomique, Castanet-Tolosan, France; 2 Laboratoire de Biométrie et Biologie Evolutive, UMR CNRS 5558, Université Claude Bernard Lyon 1, Villeurbanne, France; 3 Muséum National d'Histoire Naturelle, UMR CNRS 7179, Brunoy, France; 4 Centre de Biologie pour la Gestion des Populations, Institut National de la Recherche Agronomique, Montferrier-sur-Lez, France; 5 Office National de la Chasse et de la Faune Sauvage, Centre National d'Etudes et de Recherches Appliquées Cervidés-Sangliers, Bar-le-Duc, France; University of Sydney, Australia

## Abstract

**Background:**

Data on spatial genetic patterns may provide information about the ecological and behavioural mechanisms underlying population structure. Indeed, social organization and dispersal patterns of species may be reflected by the pattern of genetic structure within a population.

**Methodology/Principal Findings:**

We investigated the fine-scale spatial genetic structure of a roe deer (*Capreolus capreolus*) population in Trois-Fontaines (France) using 12 microsatellite loci. The roe deer is weakly polygynous and highly sedentary, and can form matrilineal clans. We show that relatedness among individuals was negatively correlated with geographic distance, indicating that spatially proximate individuals are also genetically close. More unusually for a large mammalian herbivore, the link between relatedness and distance did not differ between the sexes, which is consistent with the lack of sex-biased dispersal and the weakly polygynous mating system of roe deer.

**Conclusions/Significance:**

Our results contrast with previous reports on highly polygynous species with male-biased dispersal, such as red deer, where local genetic structure was detected in females only. This divergence between species highlights the importance of socio-spatial organization in determining local genetic structure of vertebrate populations.

## Introduction

The fine-scale genetic structure of populations, i.e. the non-random spatial distribution of genetic variation at a local scale, is strongly influenced by species-specific social structure, dispersal patterns and mating system (e.g. [1] on killer whale, [2] on brush-tailed rock wallaby). In particular, between-sex-differences in dispersal, a high level of polygyny, and strong spatial associations between relatives all enhance genetic structure [3].

In mammals, most species are polygynous [4] and natal dispersal is generally biased towards males [5]. Females are often organized into matrilines and the close spatial associations of relatives may favor cooperation and kin selection, and so enhance individual fitness [6]. This social system is thought to have evolved as the result of mate-defense mating tactics [7].

To date, most studies of fine-scale genetic structure have focused on polygynous and social species displaying male-biased natal dispersal (e.g. on red deer [8]–[10], on wild boar [11], on brush-tailed rock-wallaby [2], on common vole [12]), and much less is known about the genetic structure of solitary species (but see [6] on brown bears, [13] on a rodent: talar tuco-tuco, [14] on Canadian lynx). Contrary to most mammals studied so far, the European roe deer (*Capreolus capreolus*), living alone or in small family groups, deviates from this general pattern, exhibiting a low level of polygyny [15] and no between-sex differences in natal dispersal [16], [17]. Roe deer thus offers a unique opportunity to test whether social structure and sex-biased dispersal drive fine-scale genetic structure among large mammalian herbivores.

The aims of this study were hence to investigate the impact of this social system on population genetic structure at a local scale, by studying spatial patterns of genetic relatedness for male and female roe deer. We expected to find fine-scale spatial genetic structuring because adults of both sexes are highly sedentary. Therefore we tested the hypotheses that (1) the social structure of roe deer results in a positive correlation between relatedness and spatial proximity at a fine spatial scale, as has been observed in previously studies of ungulates and (2) the lack of a sex-bias in natal dispersal and the low level of polygyny in roe deer results in similar patterns of spatial genetic structure for the two sexes.

## Materials and Methods

### Study site and sampling

We sampled 643 roe deer from an enclosed population located at Trois-Fontaines, a forest of 1360 ha in North-Eastern France (48°43′N, 2°61′W) that has been intensively monitored by Capture-Mark-Recapture (CMR) since 1976 (for a description, see [18]). The study site and the organization of the captures are managed by the ‘Office National de la Chasse et de la Faune Sauvage’ and roe deer captures and marking were approved by the French administration (prefectural order from Paris no. 2009-014) taking into consideration the animals' welfare according to the French law. All efforts are made to minimize suffering of animals. Each individual caught was weighed, sexed, aged and the body condition was evaluated. Then, each individual was marked with plastic ear tags. During the marking (by ear piercing), a piece of ear skin tissue was taken from each animal and preserved in 100% ethanol to perform genetic analyses.

In this study, we focused on adults only (>1 year old) to avoid the sampling of doe-fawn pairs prior to weaning and potential dispersal. The individuals were captured between 2002 and 2008 using drive netting and we recorded the geographic coordinates of the forest plot (mean plot size of 8 ha) where each individual was caught.

Genomic DNA was extracted from ear tissue using a DNeasy Tissue Kit (Qiagen). DNA was then amplified by polymerase chain reaction and genotyped with a multiplex panel of 12 microsatellites [19] in a genotyper ABI PRISM 310 (Applied Biosystems). These microsatellites were characterized in different species of ungulates: IDVGA29, IDVGA8, CSSM39, CSSM41, CSSM43, BM1706, HUJ1177, BMC1009, BM848, BM757 in *Bos taurus*, OarFCB304 in *Ovis aries* and NVHRT48 in *Rangifer tarandus* [17]. Genotypic profiles were determined using Genescan and Genotyper software (Applied Biosystems) (see [20] for further details). We excluded 58 genotypes with more than 10 undetermined alleles from the analyses. Of the 585 remaining genotypic profiles, 18 genotypes were repeat-genotyped at random, with a repeatability of 97.6%. Missing data represented 0.006% of this final data set.

### Genetic diversity

We first used Micro-Checker v.4.0.7 [21] to test for the presence of any potential genotyping errors (presence of null alleles and stutters or errors caused by large allele drop-out). We then used Genepop [22] to test for linkage disequilibrium between loci and to test for deviations from Hardy-Weinberg equilibrium for each locus and globally, using the Markov chain method with 10000 iterations. We used the false discovery rate (FDR) control to correct for multiple testing, when necessary [23], using the package “qvalue” in R v.2.8.1 software [24].

### Spatial autocorrelation analyses

To assess the spatial genetic structure of male and female adult roe deer, we performed spatial autocorrelation analyses of their genotypes: we estimated a relationship coefficient among pairs of individuals belonging to the same sex and to the same *a priori* defined distance classes; for each class, random permutations of spatial locations of individuals (10000 permutations) were then used to assess deviations of the relationship coefficient from 0. Deviation from 0 means that individuals within a given distance class are significantly more (positive values) or less (negative values) related than at random. These analyses were performed with SPAGeDi v.1.2 [25]. We used distance classes in multiples of 160 m (i.e. the average radius of a forest plot). Due to its low variance, we chose the relationship coefficient *r* from Li *et al.* [26], which is corrected for sample size, bounded by [−1; +1]. In order to assess the reliability of the results obtained with this coefficient, we also performed the spatial autocorrelation analyses using three other estimators that are regularly used in similar studies: the relationship coefficients of Queller and Goodnight [27], Lynch and Ritland [28] and Moran's I statistic [29]. As advised by Hardy and Vekemans (2002) [25], we took into account only those distance classes with more than 100 pairwise comparisons, with a participation (i.e. the proportion of all individuals represented at least once in each interval) greater than 50% and a coefficient of variation of participation less than 1.

## Results

### Genetic diversity

No genotyping error was detected by Micro-Checker. A total of 585 genotypic profiles were analyzed, of which 272 were females and 313 males. The mean number of alleles per locus was 7, ranging from 2 to 20.

The values of heterozygosity expected under Hardy-Weinberg equilibrium (corrected for sampling bias) and observed heterozygosity for all loci were respectively 0.675 and 0.659. Global F_IS_ was 0.023 and heterozygote deficiency per locus was not statistically significant after FDR-control, but global heterozygote deficiency (all loci) occurred (*p* = 0.0005). There was linkage disequilibrium between some pairs of loci after FDR-control (25 out of 66 – results not shown). The loci OarFCB304 (9 pairs out of the 25 significant pairs) and BM757 (6 pairs out of 25) seemed to occur the most frequently in linkage disequilibrium of loci pairs.

### Spatial autocorrelation analyses

**Figure 1:**
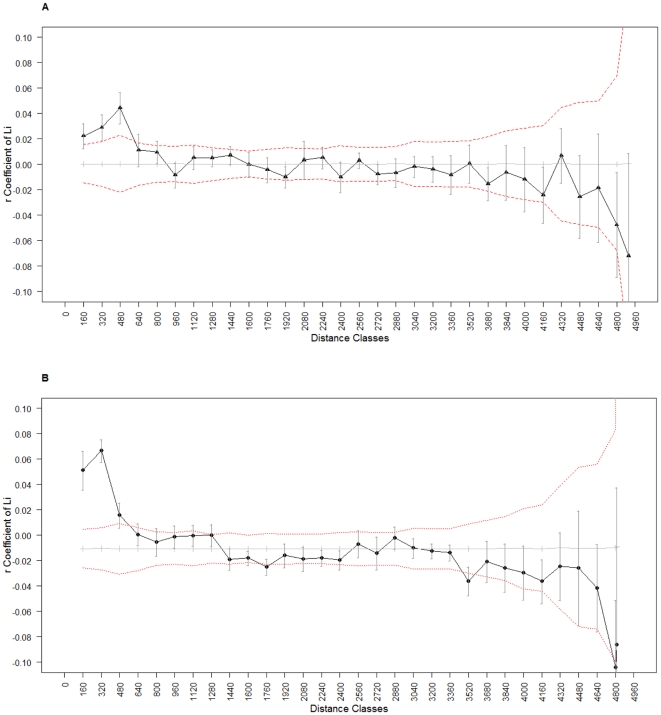
Spatial auto-correlograms of the coefficient *r* in relation to distance for pairs of individuals. We represented the estimated genetic correlation coefficient *r* of Li *et al.* (1993) (black line) in relation to inter-individual distance for pairs of females (A) and males (B), respectively. Error bars represent the standard error around *r*. The permuted 95% confidence intervals (dashed red lines) around the null hypothesis of a random distribution of individuals (average coefficients after random permutations; gray line) are also shown.

The average geographic distance between pairs of males and pairs of females were 2474 m and 2477 m, respectively. Li's average relatedness coefficient (*r*) between pairs of females and pairs of males were −0.0005±0.002 and −0.009±0.005 (mean±SE), respectively. The maximum relatedness coefficient within a distance class was 0.044±0.004 for females (between 320–480 m) and 0.069±0.009 for males (between 160–320 m). Positive *r*-values (*p-values*<0.01) occurred in the autocorrelation analysis up to 480 m for both females (Figure 1A) and males (Figure 1B). Therefore, females and males within this distance threshold are more related than expected at random, with a similar pattern of spatial genetic structure for both sexes. Similar patterns were observed with the other estimators of genetic relationships among individuals, with slight differences in the maximum distance at which spatial autocorrelation occured (Figure S1).

## Discussion

As predicted, the correlograms for both sexes were very similar and showed that genetic relatedness among roe deer is spatially structured within the Trois-Fontaines population. The similarity in the patterns of spatial genetic structure amongst individuals for males and for females is coherent with the recently reported lack of sex bias in roe deer dispersal [16], [17] and the low level of female philopatry compared to most other mammals. These results also indicate that the spatial distribution of individuals is not random: adults of both sexes tend to be located spatially close to their relatives. This is consistent with the observation that adults are highly sedentary [30] and with the low dispersal distance in our study site [17]. Although long-distance dispersal was limited by the enclosed nature of the study site, our analysis was conducted at a fine scale, hence our results should not be markedly affected. Furthermore, although some previous studies found some variability in patterns depending on the marker-based estimator used [31], our results were not affected by the type of estimator (Figures S1 A, B and C), hence our results appear robust.

The population genetic analyses revealed significant heterozygote deficiency and linkage disequilibrium. These results differ from those previously reported from the same population, but including fawns only [20]. The linkage disequilibrium and the deviation from Hardy-Weinberg equilibrium could be explained by the spatial autocorrelation amongst individual roe deer, supporting the existence of clans in roe deer populations at high density [32]. The F_IS_ value was not very large, but the significant heterozygote deficiency in the global population supports the observation of a population structured in family groups. Also, given our large data set (585 individuals), the tests were certainly powerful enough to detect even a low level differentiation in the population.

In line with most studies on large mammals, but in contrast to our results, previous studies on ungulates have revealed differing patterns of spatial genetic structure in the two sexes, with spatial genetic structure generally observed among females, but not males (Table 1). In red deer, this result was explained by strong male-biased dispersal and female philopatry [8]–[10]. Similar results of non-random spatial association among female white-tailed deer have also been observed [33]–[36], whereas males were unrelated at all distances [36], and this has been linked to strong female philopatry and the formation of matrilines. At a finer spatial scale (<100 m), Coltman *et al.* [37] also observed a non-random spatial association among individuals of the same group of a Soay sheep population (*Ovis aries*), mostly driven by female philopatry. Similarly to roe deer, the wild boar (*Sus scrofa*) displays a low level of polygyny, with female philopatry and the formation of matrilines (clans), but male-biased dispersal. The patterns of spatial autocorrelation in wild boar were also observed to be different in the two sexes (with a significant positive spatial genetic structure for females, but not males) [11].

**Table 1:** Table summarizing published studies in which spatial autocorrelation has been reported in ungulate species.

Species	Mating system and dispersal behavior	Autocorrelation patterns for sexes	Correlation coefficient	Reference
Red deer (*Cervus elaphus*)	Polygynous with strong female philopatry and male-biased dispersal	Significant positive spatial genetic structure for females but not males	Relationship coefficient of Lynch and Ritland (1999) [28]	[8]
			Kinship coefficient of Loiselle *et al.*(1995)[38]	[9]
			Kinship coefficient of Loiselle *et al.*(1995)[38]	[10]
White-tailed deer (*Odocoileus virginianus*)	Polygynous with strong female philopatry and male-biased dispersal	Significant positive spatial genetic structure for females	Relationship coefficient of Queller and Goodnight (1989) [27]	[33]
			Relationship coefficient of Lynch and Ritland (1999) [28]	[34]
			Moran's I [29]	[35]
		Significant positive spatial genetic structure for females but not males	Moran's I [29]	[36]
Wild boar (*Sus scrofa*)	Weakly polygynous with female philopatry, formation of matrilines and male-biased dispersal	Significant positive spatial genetic structure for females but not males	Kinship coefficient of Loiselle *et al.* (1995)[38]	[11]
Domestic sheep (*Ovis aries*)	Socio-structure in groups, female philopatry, formation of matrilines	Significant positive spatial genetic structure for females but not males	Relationship coefficient of Lynch and Ritland (1999) [28]	[37]
		Significant positive spatial genetic structure for females.	Wright's coefficient of relatedness estimated using pedigrees	[39]
Bushbuck (*Tragelaphus sylvaticus*)	Female philopatry, formation of matrilines and male-biased dispersal	Significant positive spatial genetic structure for females but not males	Relatedness score obtained from mitochondrial DNA analysis	[40]

In conclusion, for roe deer, the low level of polygyny [15] and the lack of between-sex differences in natal dispersal [16], [17] likely explain why we observed similar patterns of spatial genetic structure in the two sexes. This divergence between closely related species highlights the importance of socio-spatial organization in determining local genetic structure and underlines the need for further studies to understand the link between sexual selection, social structure and the evolution of sex-biased dispersal in vertebrate populations.

## Supporting Information

Figure S1 Spatial auto-correlograms of three different estimators in relation to distance. Spatial auto-correlograms for females (red lines) and males (blue lines) are represented for different estimators: Moran's I (A), Queller and Goodnight (B) and Lynch and Ritland (C). Error bars represent the standard error around the estimated genetic correlation coefficients. The permuted 95% confidence intervals (dashed lines, in red for females and in blue for males) around the null hypothesis of a random distribution of individuals (average coefficients after random permutations; gray line) are also shown.
(1.39 MB TIF)
