# Supplementary figures and images for: No Difference between the Sexes in Fine-Scale Spatial Genetic Structure of Roe Deer
Source: PLoS One. 2010 Dec 28;5(12):e14436. doi: 10.1371/journal.pone.0014436 (PMC3010998; doi:10.1371/journal.pone.0014436)

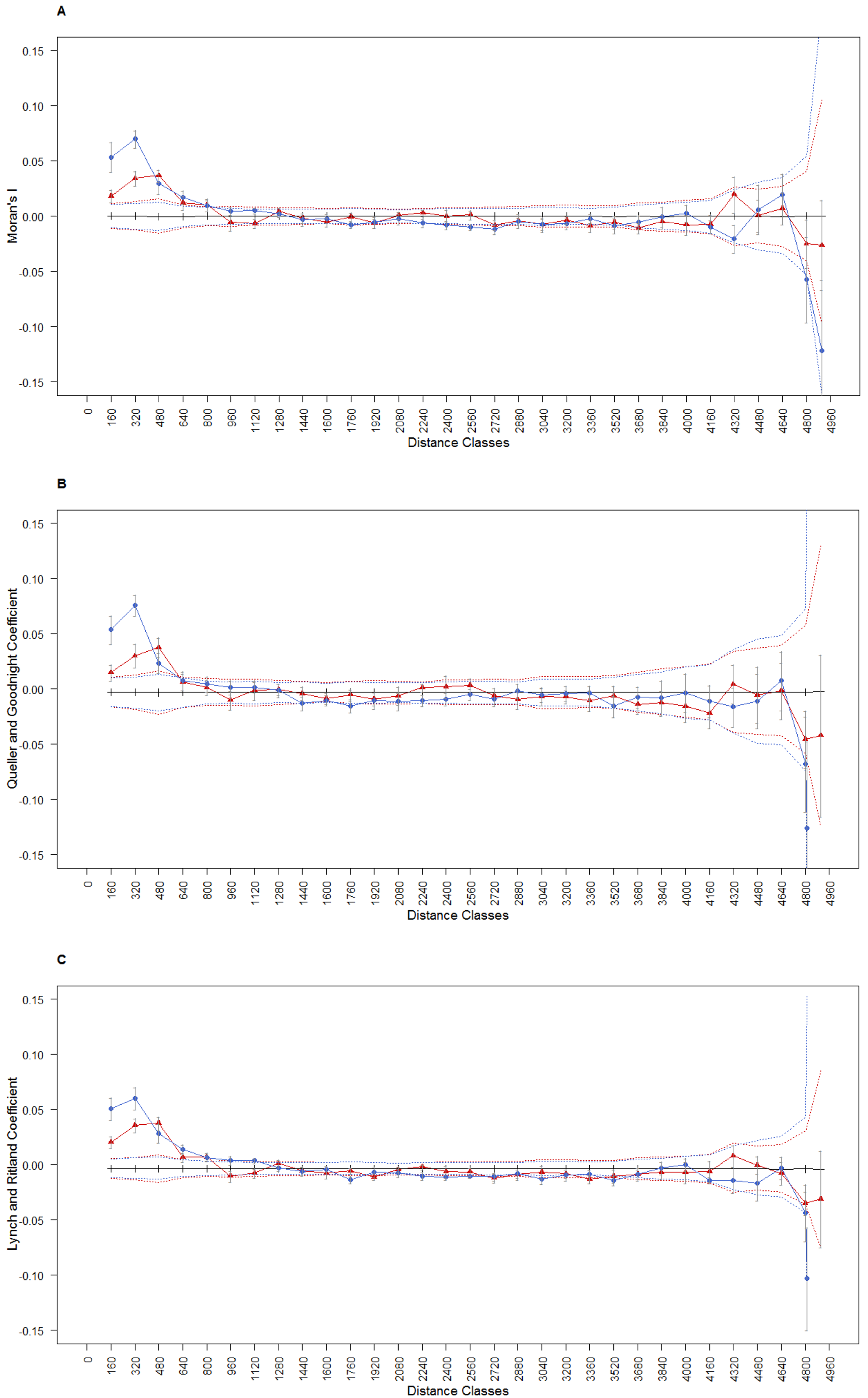

Supplement: Figure S1 — Spatial auto-correlograms of three different estimators in relation to distance. Spatial auto-correlograms for females (red lines) and males (blue lines) are represented for different estimators: Moran's I (A), Queller and Goodnight (B) and Lynch and Ritland (C). Error bars represent the standard error around the estimated genetic correlation coefficients. The permuted 95% confidence intervals (dashed lines, in red for females and in blue for males) around the null hypothesis of a random distribution of individuals (average coefficients after random permutations; gray line) are also shown. (1.39 MB TIF) [file pone.0014436.s001.tif]
